# Hyaluronic Acid Allows Enzyme Immobilization for Applications in Biomedicine

**DOI:** 10.3390/bios12010028

**Published:** 2022-01-07

**Authors:** Jackie Arnold, Jordan Chapman, Myra Arnold, Cerasela Zoica Dinu

**Affiliations:** 1 Department of Chemical and Biomedical Engineering, Benjamin M. Statler College of Engineering and Mineral Resources, West Virginia University, Morgantown, WV 26505, USA; jra0031@mix.wvu.edu (J.A.); jschapman@mix.wvu.edu (J.C.); 2 Department of Sociology and Anthropology, Eberly College of Arts and Sciences, West Virginia University, Morgantown, WV 26505, USA; mba0024@mix.wvu.edu; 3 Department of Business Incubator, John Chambers College of Business and Economics, West Virginia University, Morgantown, WV 26505, USA

**Keywords:** enzyme, biomedicine, hyaluronic acid, immobilization, bioengineering, catalysis, nanomaterials

## Abstract

Enzymes are proteins that control the efficiency and effectiveness of biological reactions and systems, as well as of engineered biomimetic processes. This review highlights current applications of a diverse range of enzymes for biofuel production, plastics, and chemical waste management, as well as for detergent, textile, and food production and preservation industries respectively. Challenges regarding the transposition of enzymes from their natural purpose and environment into synthetic practice are discussed. For example, temperature and pH-induced enzyme fragilities, short shelf life, low-cost efficiency, poor user-controllability, and subsequently insufficient catalytic activity were shown to decrease pertinence and profitability in large-scale production considerations. Enzyme immobilization was shown to improve and expand upon enzyme usage within a profit and impact-oriented commercial world and through enzyme-material and interfaces integration. With particular focus on the growing biomedical market, examples of enzyme immobilization within or onto hyaluronic acid (HA)-based complexes are discussed as a definable way to improve upon and/or make possible the next generation of medical undertakings. As a polysaccharide formed in every living organism, HA has proven beneficial in biomedicine for its high biocompatibility and controllable biodegradability, viscoelasticity, and hydrophilicity. Complexes developed with this molecule have been utilized to selectively deliver drugs to a desired location and at a desired rate, improve the efficiency of tissue regeneration, and serve as a viable platform for biologically accepted sensors. In similar realms of enzyme immobilization, HA’s ease in crosslinking allows the molecule to user-controllably enhance the design of a given platform in terms of both chemical and physical characteristics to thus best support successful and sustained enzyme usage. Such examples do not only demonstrate the potential of enzyme-based applications but further, emphasize future market trends and accountability.

## 1. Introduction

Working from the cellular to organismic level, each of the physiological systems that govern the biological world requires and/or benefits from the inherited abilities of enzymes, proteins that catalyze a wide variety of unique and specific processes. Such biocatalysts decrease the necessary energy input to facilitate biological reactions with incredible selectivity sparking interest for synthetic implementation as active candidates in a variety of engineered applications. There are, however, limitations that accompany the benefits that enzymes bring to the scientific community, most associated with their pH and temperature-induced fragility, as well as unprofitable user-controllability, shelf-life, and scale-up systematic integration, just to name a few.

To mitigate such limitations, enzyme immobilization has become a prevalent approach. This review provides an overview of current implementation of enzymes in synthetic applications with focus on biofuel [1,2,3], textile [4,5,6,7,8], detergent [4], waste management [8,9], and food and drink industries [10,11] respectively. Subsequently, particular attention is directed towards enzyme immobilization using hyaluronic acid (HA) scaffolds, a polysaccharide consisting of alternating β-1,4-D-glucuronic acid and β-1,3-N-acetyl-D-glucosamine rings known for its high biocompatibility, biodegradability, viscoelasticity, and hydrophilicity [12,13,14], to thus help emphasize enzymes’ implementation in biomedical applications. Discussions are in support of specific applications in drug delivery [15,16,17], tissue engineering including wound healing [18,19,20] and biosensors [21,22,23,24], respectively. Challenges associated with enzymes exploitations in synthetic environment are analyzed. Lastly, potential for marketability and perspectives on consumer usage are provided to thus capitalize on the extended implementation that enzyme-based products could have.

## 2. Brief Description of Applications of Enzymes in Consumer Industries

### 2.1. Enzymes in Biofuels

Selective depletion of nonrenewable resources of fossil fuels as well as the high climate impact that processing of such resources leads to are providing means for alternative integration of enzymes for and in biofuels production respectively. Such integration is foreseen to reduce the environmental factor (E-factor; a known measure of the mass of undesired non-water waste produced by a chemical process per mass of its desired product [25]) as well as the dependence on imported fuel [26,27].

Advanced biochemical processes using enzymes are seen to improve biofuel production efficiency under less energy intensive requirements and possibly with low costs. Such biochemical processes frequently either rely on or employ focused genetic engineering approaches, based on the use of recombinant DNA technology especially known to produce large quantities of recombinant enzymes, or enzymes that are efficient when used in the synthetic production of biofuels. Moreover, such approaches have proved to lead to recombinants with high resistance capability toward contaminations and/or to highly thermo-stable forms. For instance, the fatty acid biosynthesis pathway as well as the triacylglycerol (TAG) assembly of algal enzymes have been manipulated to demonstrate increases in the oil content of biofuel-producing algae. Over-expression of key enzymes including but not limited to acyl-CoA synthetases (ACSs) and diacylglycerol acyltransferase (DGAT), respectively, enabled a more cost-efficient biofuel with operative production processes being realized by increasing the ability of the microalgae to produce lipids and subsequently fuels [1,2]. The method led to a reduced impact environmental impact and a reduction in cost relative to the traditional and more expensive non-modified microalgae biofuel production systems [2].

Complementary, Council of Scientific and Industrial Research (CSIR)—Institute of Minerals and Materials Technology in India has developed a database highlighting the enzymes involved with the lipid biosynthetic pathway (i.e., the process by which microalgae produces bioactive lipids such as triacylglycerols [28]), with microalgal enzymes being classified by their length, hydrophobicity, amino acids composition, subcellular location, gene ontology, Kyoto Encyclopedia of Genes and Genomes (KEGG) pathway, orthologous group, Pfam domain, intron-exon organization, transmembrane topology, and secondary/tertiary structural data, respectively [1]. Genetic and physical characteristics of such enzymes were subsequently compiled for a concise source integration and optimized enzyme utilization [1]. Analysis by Misra et al., for instance, showed higher photosynthetic efficiency and biomass production than starch-based and lignocellulosic plant species currently used for biofuel-purposed lipid production.

Production of hydrogen ($H_{2}$) for a comparatively clean and renewable option was also attempted through the usage of enzyme-facilitated metabolism algal functions [3,29]. Among current methods for $H_{2}$ production are pyrolysis [30,31,32] and gasification [33,34] of carbon-based fossil fuels, sewage sludge, biomass as well as steam reforming of natural gas [35,36], and electrolysis of water [34,37,38], respectively. However, such methods are limited by the profitability in the $H_{2}$ yield, its storage and distribution capabilities, severe reaction conditions required at its implementation, as well as related equipment fragility and energy input necessities [34,35,39] respectively. It was thus predicted that within the next 30 years, the $H_{2}$ production would greatly help shift the global energy system (GES) integration towards more sustainable energy system (SES) [37], whereby greenhouse gas emissions will approach net zero [39]. Though enzymes do come with a significant production cost, their potential for genetic modification and long-term economic profit have been considered to overcome the challenges associated with traditional methods for $H_{2}$ production [40]. Meanwhile, the production costs of current commercial ways can only be pacified with the adoption of new theory and methodology altogether [33].

**Figure 1:**
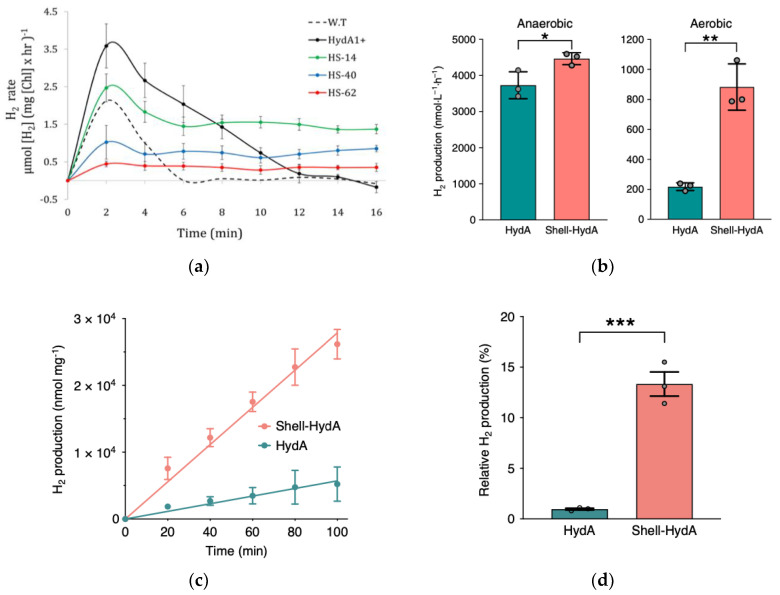
(**a**) HydA-SOD and clones of the complex rates of reaction over time. Reprinted with permission from ref. [3]. 2019 Springer Nature. (**b**) In vivo testing of anaerobic and aerobic pathways of $H_{2}$ production for Shell-HydA relative to free HydA enzyme (* signifies *p* = 0.0352 and ** signifies *p* = 0.0018). Reprinted with permission from ref. [41]. 2020 Springer Nature. (**c**) In vitro enzyme-mediated $H_{2}$ production over time of Shell-HydA composite relative to free HydA. Reprinted with permission from ref. [41]. 2020 Springer Nature. (**d**) Anaerobic relative $H_{2}$ production for Shell-HydA and lone HydA found through in vitro testing of activity following 24 h of exposure to O_2_ at 4 °C (*** signifies *p* = 0.0009). Reprinted with permission from ref. [41]. 2020 Springer Nature.

In this regard, research by Ben-Zvi et al., utilized algal biocatalysts within the *C. reinhardtii* for the natural photoproduction of H_2_ [3,29]. Authors have focused on hydrogenase, an enzyme capable of catalyzing the production of $H_{2}$ with incredible activity and efficiency [41,42], and especially [Fe-Fe]-hydrogenase [3], the well-known enzyme used for catalytic H_2_ turnover [41]. [Fe-Fe]-hydrogenase (HydA) and superoxide dismutase (SOD) were fused through genetic code sequencing to form a complex that was shown to lead to increased ability for catalyzed $H_{2}$ production, with the Hyd-SOD fusion protein being subsequently cloned. Cloned complexes possessed inherent ability to continuously and easily form $H_{2}$; Hyd-SOD clones also saw greater prolonged H_2_ photoproduction relative to the D66 *C. reinhardtii* wild type and a strain exhibiting the HydA1 gene, all when evaluated following the natural production burst initially experienced (Figure 1a) [3]. The presence of the hydrogenase enzymes was imperative for the sustainable $H_{2}$ generation since the catalysts reduced the surplus electrons to form a useful energy carrier; moreover, they helped outcompete electron loss to the Calvin–Benson–Bassham (CBB) cycle [3]. Furthermore, the metalloenzyme SOD played an important role in increasing continuous and steady $H_{2}$ production as well as likely addressing the limitation of HydA in its sensitivity to oxygen (O_2_), a product of simultaneous water splitting [3].

To protect the hydrogenase from O_2_ byproducts, Li et al., proposed the use of carboxysome shells, “virus-like” bacterial organelles that were shown to provide favorable microenvironments. Analysis showed increased ability for H_2_ catalytic performance especially when *Escherichia coli* were used as genetically modified hosts to allow encapsulation of [Fe-Fe]-hydrogenases and ferredoxin isolated from *C. reinhardtii* algae [41]. The potential of such complexes was proven in the increased enzymatic resistance to O_2_ as well as increased amount of $H_{2}$ produced with respect to free HydA, especially under aerobic conditions in vivo (Figure 1b). Moreover, difference in H_2_ production between the shell-HydA composite and free HydA increased over 100 min when tested in vitro, thus proving a sustained benefit to the enzyme technology (Figure 1c). Additionally, purified shell-HydA complex maintained 13.33 ± 2.06% of the activity following O_2_ exposure when compared to only 0.97 ± 0.15% activity observed for the free enzyme (Figure 1d). The encasement allowed for the hydrogenase complex to be more selectively permeable to substrates thus leading to improved enzymatic activity [41].

In addition to their O_2_ susceptibilities, Dolui et al., considered their water solubility and acid stability limitations in applications involving H_2_-producing hydrogenase enzymes. Utilizing cobaloxime complexes consisting of six derivatives with varying side chains, an enzyme-inspired, multicomponent outer coordination sphere was studied as a means to decrease the effect of O_2_ on hydrogenase activity as well as to increase enzyme’s rate of reaction, solubility in water, and resistance to acidic environments respectively. [42]. Authors demonstrated that such enzyme-based complexes led to fast, energy efficient, and/or stable catalytic functionality for more profitable integration in enzyme-based renewable energy sector [42].

Lastly, in a study by Zhang et al., a 13-enzyme pathway converted starch and water into $H_{2}$ with an improved production yield over previously explored natural, anaerobic fermentation methods, among others. Such improvement increased from the theoretical value of 4 H_2_/glucose to 12 H_2_/glucose with biocatalyst cascade application. The use of enzymes benefited from the mild reaction conditions (30 °C and atmospheric pressure), as well as low production costs (~USD 2/kg $H_{2}$), and a high energy-density carrier starch (14.8 H_2_-based mass %)” [39].

### 2.2. Enzymes for Waste (Plastic and Chemical) Management

Aside from making an impact on the environment and on the growing demands associated with renewable energy and sustainable resources creation, enzymes have also been investigated for their ability to breakdown plastic [9,43,44,45,46] and chemical waste [47,48,49].

Briefly, current techniques for plastic management consist of chemical or physical recycling [43,45], reusage of existing plastic materials, or plastic removal to dedicated landfills [9,43,45] for incineration [50]. However, such techniques are simply inadequate in their capacity to remove the environmental, health, and planetary life risk [43,45,50], namely water pollution, harm to flora and fauna, toxicity, and greenhouse gas emissions, just to name a few of such effects. Further, in the chemical depolymerization of poly(ethylene terephthalate) (PET) main constituent synthetic polymer commonly used in the packaging [51] and textile [52] industries [9] or the mechanical recycling of such materials for instance, there is a significant loss to processing costs and resultant byproduct properties, respectively [9]. As such, plastics’ inability to degrade quickly and safely requires a complete shift in the methods used for their disposal [43,50].

In a study by Austin et al., for instance, PET-ase enzymes were employed to decompose the non-biodegradable PET. Biomimetic analysis identified that *Ideonella sakaiensis* secretes PET-ase to harvest the carbon available within environmentally harmful PET sources. Such sources include single-use beverage bottles, clothing, packaging, and carpeting. With the use of PETase, PET was primarily converted to mono(2-hydroxyethyl) terephthalic acid (MHET), though terephthalic acid and bis(2-hydrocyethyl)-TPA are also produced during catalysis [9,43]. Studies also showed that by mutating the PETase enzyme, the percent crystallinity change as well as MHET and TPA production were increased in comparison to those of the natural PETase enzyme [9].

**Figure 2:**
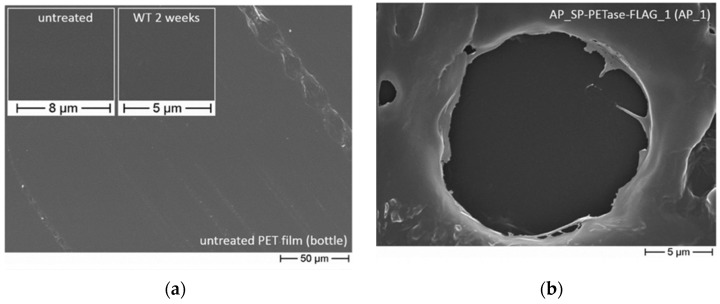

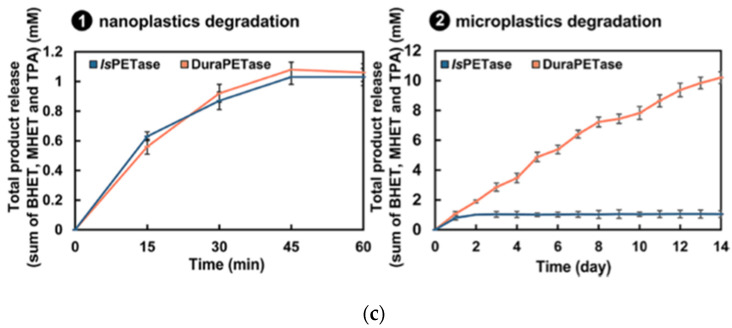
(**a**) Untreated PET film, as seen via scanning electron microscopy, and representative of the standard, smooth surface of a PET-based bottle film. Reprinted with permission from ref. [43]. 2019 Springer Nature. (**b**) Scanning electron microscopy analysis of PET bottle film following five weeks of exposure to and subsequent degradation by cells expressing ${\mathrm{AP}}_{\mathrm{SP}}\_{\mathrm{PETase}}^{R280A}-\mathrm{FLAG}$; degradation is shown as a sign of wear. Reprinted with permission from ref. [43]. 2019 Springer Nature. (**c**) Plots corresponding the total sum of the concentration of PET degradation products with time following the respective nanoplastics’ and microplastics’ incubation with *Is*PETase and DuraPETase. Reprinted with permission from ref. [45]. 2021 American Chemical Society.

Among the most significant locations increasingly ailed by plastic waste are the ocean ecosystems [43,46,50,53,54]. To help dampen this astounding issue, Moog et al., utilized *Phaeodactylum tricornutum* microalga to produce and secrete the PETase enzyme for the degradation of both PET and polyethylene terephthalate glycol (PETG) in saltwater analysis showed that such PETase possessed degradation activity against both PET and PETG, including under moderate conditions and in saltwater [43] (Figure 2a,b respectively). While *Ideonella sakaiensis* bacteria was considered for its ability to produce the PETase, this specie showed limited performance in ocean-like environments presumably due to its instability and inability to survive in such aqueous habitats [43,55].

Further, Cui et al., applied the greedy accumulated strategy for protein engineering (GRAPE) to improve the robustness of a PETase isolated from *Ideonella sakaiensis* (*Is*PETase) [45]. Both DuraPETase and *Is*PETase caused total nanoplastic degradation following treatment for 1 h at 37 °C, according to collected high-performance liquid chromatography data (Figure 2c (1)). As for microplastics, DuraPETase was able to breakdown the larger particles to close to completion within two weeks, displaying water treatment activity much greater than those of *Is*PETase (Figure 2c (2)).

PETase was also active towards low and high-crystallinity plastics such as bottle-grade PET; further, monohydroxythyl terephthalate hydrolase (MHETase), exo-PETase and bis-(2-hydroxyethyl)terephthalate (BHET)ase were also shown to catalyze PET film degradation [46]. Moreover, MHETase completed the biodegradation of PET into its basic components, with hydrolysis activities of MHETase toward PET films showing increased potential to impact PET degradation as a multifaceted approach for efficient waste management [46].

Coppella et al., highlighted the use of parathion hydrolase against organophosphate wastes for insecticide-containing waste degradation [47]. Kinetics observation revealed the rapid enzymatic reaction followed saturation kinetics with authors concluding that parathion hydrolase enzyme was able to hydrolyze coumaphos and potasan under conditions that closely simulated those expected in the field [47].

In accordance with the waste generated via US nuclear weapons downsizing around 1999, Vanderberg et al., further demonstrated enzymes’ capability to treat hazardous radiologically contaminated heterogeneous paint-stripping waste. Authors showed that cellulase degraded cellulose-based organic materials with analysis revealing that cellulose bulk volume was decreased by 80% following cellulase digestion, with the resultant radioactive sugar products and additional hazardous metals being easily removed by polymer filtration methods [48].

### 2.3. Enzymes in Detergent and Textile Industries

Biocatalysts are also commonly used in the detergent and textile industries [4,5,6,8]. Kumari et al., for instance, showed how leaf enzymes could serve as highly stable catalysts for stain removal, biowashing, and biopolishing tasks with mannanase, leaf lipase and cellobiohydrolase all being tested against common enzymes used in the detergent industry (i.e., nineteen products in liquid and powder forms, including twelve endoglucanases, five lipases, and two mannanases) [4].

**Figure 3:**
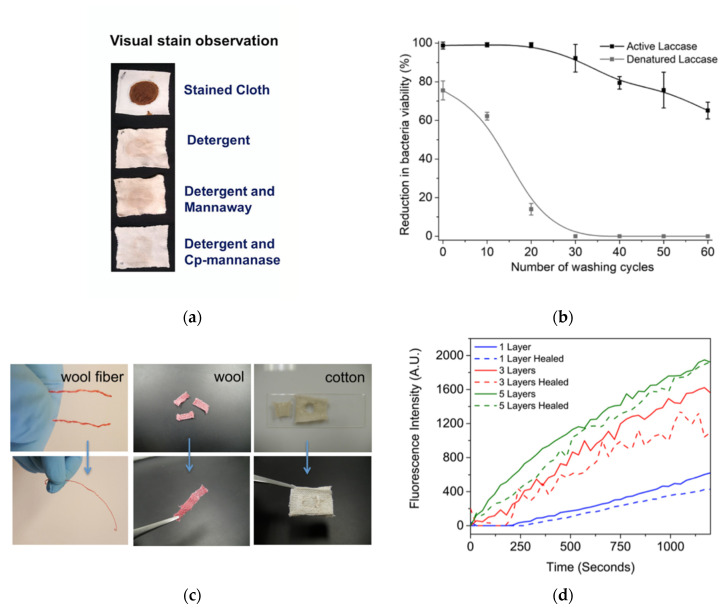
(**a**) Observation of chocolate stain removal as a result of enzyme and non-enzyme containing detergent washes. Reprinted with permission from ref. [4]. 2019 The Authors. (**b**) Comparison of active and denatured laccase enzymes in their antibacterial capacity, as represented by percent reduction in bacterial viability. Reprinted with permission from ref. [5]. 2018 Elsevier. (**c**) Visual representation of wool and cotton textile samples before and after self-healing. Reprinted with permission from ref. [6]. 2016 American Chemical Society. (**d**) Efficiency of enzymatic activity, as represented by fluorescence intensity, of multilayer systems both before and after the self-healing process. Reprinted with permission from ref. [6]. 2016 American Chemical Society.

All the enzymes sourced from crude leaf extracts, apart from Cp-Eg1, were considered stable following biopolishing, biowashing, and stain removal as well, while Cp-lipase and Cp-mannanase were increasingly thermostable relative to their commercial counterparts. In contrast to commercially used Novoprime 868, Cp-Eg1 and Cp-CelD, the isolated enzymes also proved capable of uniform indigo dye removal from denim samples, without affecting the fabric quality. Moreover, Cp-Eg1 and Cp-CelD displayed equal and slightly improved efficiency in the biopolishing process, respectively, while Cp-mannanase presence increased the effectiveness of detergent-based stain removal (Figure 3a) [4].

Further, enzymes were used for antibacterial textiles formation [5,7]. Antibacterial gowns and linens theoretically help prevent the growth of bacteria resistant to the antibiotics currently in use in hospitals settings [5,56]. ZnO nanoparticles (NPs) were for instance used by Salat et al., to break down the membranes of Gram-positive and Gram-negative bacteria through production of reactive oxygen species (ROS). Analysis also showed that ZnO NP deposition via active laccase led to increased and sustained antibacterial performance when compared to similar coatings developed with inactive laccase enzyme and using crosslinking respectively. However, a significantly less number of ZnO NPs was able to be initially deposited, and a negligible amount of ZnO, and therefore, reduction of bacteria viability was detectable on the textile surface following 30 washes thus representing the difference in the use of operative enzyme vs. without (Figure 3b) [5].

Moreover, Polak et al., used fungal laccases for synthesizing a phenazine-based dye with antibacterial properties. Resulting dye contained 10-((2-carboxy-6-methoxyphenyl)amino)-11-methoxybenzo[a]phenazine-8-carboxylic acid decreased *Staphylococcus aureus* growth, a human pathogen known to develop new clones with a multitude of antibacterial resistances [57] leading to bacterial infections in the skin, soft tissue, bone, bloodstream, and respiratory tract of human patients [58] respectively. The dye production via *Cerrena unicolor* protein catalysis was not only bioactive but was also more sustainable, less toxic, less mutagenic, and easier to replicate when compared to chemical approaches that require additional and harmful coupling agents and additives including but not limited to benzene, boron tribromide (${\mathrm{BBr}}_{3}$), and dimethylformamide (DMF) [7].

Further, Gaddes et al., applied urease as a proof-of-activity enzymatic component to self-healing textiles formed using versatile polyelectrolyte layer-by-layer (LBL) films also notably containing squid ring teeth (SRT) proteins that lend the films the ability to self-adhere in an environmentally friendly, cost-efficient, feasible, and sustainable, large-scale way [6]. The coating design was envisioned to lead to extension of lifespan for both woven and nonwoven materials through the ability of the textiles to self-repair damage such as scratches, holes, or rips (Figure 3c). The number of multilayers applied was a significant contributor to the enzymes’ activity with activity increasing between 1, 3, and 5 layers, respectively (Figure 3d). Regardless of the number of layers deposited onto the cloth, there was also only slight loss of this catalytic action following sample repair (Figure 3d) [6].

Lastly, Blánquez et al., showed that bacterial laccases, particularly SilA laccase, decolor and detoxify Acid Black 48, Acid Orange 63, Reactive Black 5, Orange II, Tartrazine, Azure B, Indigo carmine, and Cresol red. Such dyes are known to remain stable and reactive in sewage plants and eventually rivers following chemical, light exposure, and microbial degradation treatments [8] and can induce toxic, carcinogenic, mutagenic, and teratogenic effects [8,59]. Biologically based treatments offered eco-friendly, cost-competitive, and waste-lacking options to approach this issue [8,59].

### 2.4. Enzymes in the Food Production and Preservation Industry

Applications of enzymes in the preservation industry consider the prominent economic and health issues in a world of exponentially expanding population with simultaneous population aging [10,60]. Studies showed that humans are so much as transitioning into an era of malnutrition due to the global issue of unsustainable food sourcing, as affected by food supply, waste, and negative environmental impact [61].

Proteolytic and chitinolytic enzymes demonstrated the ability to inhibit microorganism growth and survival that would otherwise cause fruit and vegetable spoilage [10].

In beer production, Lei et al., showed that enzyme addition to high gravity worts at the beginning of the fermentation process led to increased brewing capacity, ethanol recovery, and product stability, as well as a decrease in the cost of energy and labor [62].

Moreover, Xiao et al., considered a chemoenzymatic approach to synthesizing human milk oligosaccharides (HMOs) in the production of a library of 31 characterized HMOs [63]. Further, El-Salam et al., focused on the ability of protease from the bacterial strain *Lactobacillus plantarum*, both in the form of crude enzyme extract and purified enzyme, to improve the properties of the soft, white Domiati cheese throughout the ripening and storage periods [64].

The above reports highlight that exploration of enzymes in food and beverages industries, or related branches, offers the possibility for increased production [63] while enhancing attributes such as flavor [64]. Benefits of formulating and understanding HMOs, for example, include expansion of their implementation in formulas, which thus enables improved prebiotic, gastrointestinal, inflammatory and immune response and promote brain and cognition development effects for infants, especially when considering the studies of concentrations of HMOs available through human lactation [63,65]. Lastly, as shown by El-Salam et al., protease enzymes could make up 60% of the sales of enzymes worldwide, thus representing an impactful sector of industry [64].

## 3. Challenges and Perspectives for Enzymes Applications: A Case Study of Enzymes Integration in Biomedical Engineering

Studies listed previously reveal a brief summary of specific applications of enzymes in few different industrial sectors and emphasize the advantages of such systems as associated with lack of hazardous waste, diverse and growing functionality, assistance toward high reaction and increased product formation rates, as well as the ability to quickly and easily be altered through chemical, and/or physical methods. Analyses included above also identify that enzyme-based biodegradation for instance, eliminates any contaminated or uneasily managed byproducts or volatile metals at fairly cost-effective strategies [48] while further increasing our ability to address the global detriment different types of waste causes [45].

Many of these enzyme technologies applied enzymes in their native form, wherein a solution of free enzyme was added into a given process to form a product. While this form of enzyme applications has advantages including easy access of substrates and easy release of products in the catalysis process respectively, there are limitations to the transposition of enzymes from their natural state and environment respectively when various large-scale innovations are considered. Namely, enzyme implementations can have a short shelf-life, can lack user controllability, and their assisted process can be difficult to scale-up. Enzymes themselves can also be incredibly fragile when placed in non-natural conditions, with small changes in temperature and pH for instance known to reduce their applicability, shelf life, and cost efficiency. Moreover, the benefits listed above are also challenged by reduced profitability, continuous challenges associated with separation of enzymes’ from resulting products, sustained enzyme activity over extended periods of time or cycles of production, inhibition by reactants and/or products present in a given reaction mixture, as well as reduced enzyme shelf life or ability to control their functionality in extended operational conditions otherwise meant to accommodate an evolving environmental challenge or profitability.

One developing approach to reduce such limitations is through enzyme immobilization. In particular, enzyme immobilization is seen as a viable means to provide greater user controllability through specific localization strategies and control of enzymes’ catalytic activity using physical [66] or chemical [67] bounding to or within synthetic platforms including but not limited to hydrogels [17,18,19,20], particles [15,16,22], nanotubes [23], or frameworks [66]. Meanwhile, the recyclability of enzymes can also increase through such immobilization approaches, thus resulting in their repeatable use and reduction in application costs [66].

The remainder of this review explores various enzyme immobilization strategies and provides specific examples of how user-designed enzyme conjugates could offer increased integration in biomedical field for namely drug delivery, tissue engineering, and biosensing applications, just to name a few. Approaches will be focused on the use of hyaluronic acid (HA), a naturally occurring polysaccharide consisting of alternating β-1,4-D-glucuronic acid and β-1,3-N-acetyl-D-glucosamine rings. HA is known for its high biocompatibility, biodegradability, viscoelasticity, and hydrophilicity [12,13,14]. These attributes lend HA relevance in biomedicine, particularly for in vivo enterprises. This compound is easily linked to the development of amphiphilic complexes with strong immobilization functionality in addition to its ability to breakdown in governable fashions and periods of time, as beneficial in drug delivery, tissue engineering, and wound healing processes [15,16,17,18,20]. Particularly in the biosensing sector, HA’s biocompatibility and moisture-retaining properties offer anti-fouling advantages to sensing composites; HA has also shown to improve electrical conductivity for biosensors in which it is applied [21,22,23]. HA has high market potential with current analysis projecting roughly doubling incomes (i.e., an increase in the size of the HA market from USD 9.6 billion in 2020 to USD 16.6 billion in 2027) representative of an expected compound annual growth rate (CAGR) of 8.1% [68].

### 3.1. Drug Delivery

**Figure 4:**
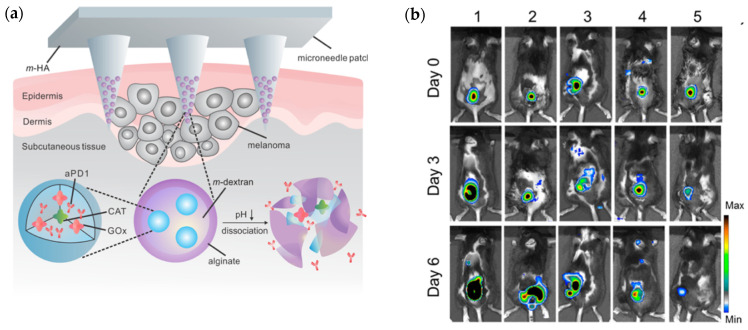

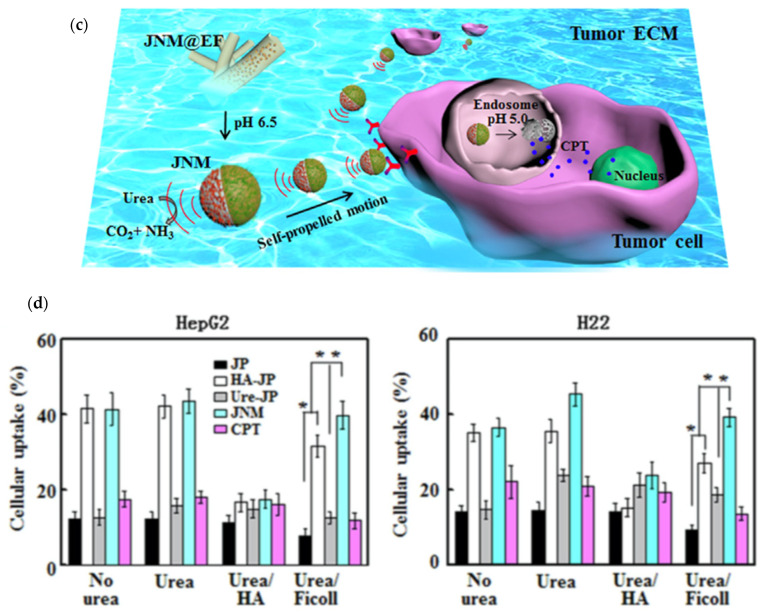
(**a**) Design schematic of degradable HA microneedle patch for the site-specific delivery of anti-cancer drugs to melanoma cells. Reprinted with permission from ref. [15]. 2016 American Chemical Society. (**b**) Tumor observation via bioluminescence imaging on day zero, day three, and day six of the experimental treatment trial performed by Wang et al., with treatments 1-5 discerning melanoma untreated and treated with MN-GOx, free aPD1, MN-aPD1, and MN-GOx-aPD1, respectively. Reprinted with permission from ref. [15]. 2016 American Chemical Society. (**c**) Schematic representation of the movement of designed nanomotors through the ECM of a tumor before intracellular dissolution for the release of cytotoxic CPT as a mean of cancer therapy. Reprinted with permission from ref. [16]. 2019 Elsevier. (**d**) HepG2 and H22 cellular uptake percentages for JPs, HA-JPs, urease-JPs, JNMs, and free CPT, respectively, in media without urea, urea alone, urea with HA, or urea with Ficoll (* signifies *p* < 0.05). Reprinted with permission from ref. [16]. 2019 Elsevier.

Enzyme immobilization has the potential to serve as targeted, effective, and efficient system that aids in drug delivery while eliminating possible unwanted biological entities, reactions, or processes associated with drug adsorption and metabolization at a tumor site [16]. For instance, enzymes glucose oxidase (GOx) and catalase (CAT) were used as components to a microneedle (MN) patch for a minimally invasive transdermal melanoma treatment (Figure 4a). The nanoparticles placed at the tips of the microneedles were spherical in shape and had an average hydrodynamic size of 250 nm. The patch was formed from HA crosslinked with N,N-methylenebis (acrylamide) (MBA) under photo initiator and UV-initiated polymerization respectively, and had a 9 × 9 mm^2^ area containing 225 cone-shaped MNs. Encapsulated GOx converted glucose to gluconic acid, producing an acidic environment that promoted the gradual release of anti-programmed death-1 (aPD1) previously encapsulated within dextran nanoparticles (NPs) to be used for skin cancer immunotherapy. Functionally, aPD1 was shown to help negate the negative effects to immunity caused by programmed death-1 receptors of T-cells [15].

Following a month of storage, the bioactivity of the MN patch system was maintained at 90%. The gradual release of aPD1 occurred over the course of three days. Increased T-cell infiltration was also observed in patch-treated tumors when compared to the control group, and most importantly, mice treated with the fully developed patch saw an increase in antitumor activity relative to mice treated with free aPD1, while tumor growth was significantly inhibited in mice treated with MN-GOx, free aPD1, MN-aPD1, and MN-GOx-aPD1, respectively(Figure 4b). Furthermore, there was no significant inflammation observed; mice saw increased lifespans, with 40% survival after 40 days following aPD1-GOx-MN treatment and about 70% of T-cell promoting anti-CTLA4 antibody and aPDI treated mice being cancer-free beyond 60 days when the MN patch was used. The slow and sustained release over three days for the HA-based MN complex, in contrast to the diffusion away from the tumor sight observed following three days for free aPD1, leant a low cost of administration for such therapy with only one administration being necessary to produce the desired immune responses within the cancerous mice tested [15].

Complementary, Chen et al., described a self-propelling cancer drug delivery system in which HA and urease were placed on opposing hemispheres of a Janus nanoparticle (JP) consisting of a mesoporous silica nanoparticle (MSN) half-coated with Au with an embedded hydroxyapatite (Hap) core and loaded with chemotherapeutic camptothecin (CPT) (Figure 4c). Such nanomotors were immobilized onto electrospun fiber fragments (JNM@EF) and administered directly to a local tumor area in cancer-bearing mice where the slightly acidic nature of the tumor matrix was enough to cause a sustained release of the JNMs from the EF platforms. Analysis showed that HA allowed targeted delivery and uptake of the released JNMs into tumor cells, while the enzyme served as a power source that projected the drug carrier forward through the ECMs of the tumor. Further, the acidic intracellular environment of cancerous tumors (pH 5.0) provided a controlled release mechanism for CPT that remained unfazed by changes in the pHs of physiological or even tumor matrix conditions, valued at 7.4 and 6.5, respectively [16].

The enzymatic propulsion mechanism was considered an improvement over those approaches based on the conversion of mechanical energy under externally applied magnetic and electric fields, namely due to an increase in user-controllability. Undesired release of CPT into the ECM was quantified at only about 8%, conferring robustness to the approach as well as to the nanocarriers prior to their uptake by the tumor cell. The fully developed JNM@EF, EF/JNM, and phosphate buffered solution (PBS) control treatments resulted in ~14.7, ~53, and ~88% of ki-67-positive cells detected in tumors at 36-, 27-, and 16-day lifespans reached by over 50% of mice tested, respectively. As shown in Figure 4d, the addition of an HA coating to the JP surface also increased the cellular uptake substantially in HepG2 and H22 cells, especially in media not containing urea, containing urea, or containing urea and Ficoll respectively. The increased uptake was relative to JPs without HA or urease coatings, urease-JPs, and free CPT respectively, with the only other drug carrying complex with greater percentages than HA-JPs being the complete JNM complex characterized with one hemisphere of HA and the other coated with urease enzyme, in all tested media and for both tested cell types respectively [16].

Moreover, in a study by Montanari et al., bovine serum amine oxidase (BSAO) enzyme was immobilized within an injectable HA-based nanohydrogel (NH) to be used for treatment of melanoma cancer. Such self-assembled amphiphilic hydrogels were formulated upon the covalent interaction between HA and cholesterol, under sonication and in water, with enzymes being conjugated via carbodiimide crosslinking. As the gel-loaded source of melanoma treatment, BSAO led to the formation of cytotoxic compounds including hydrogen peroxide and a series of aldehydes resulted from the catalysis of polyamines. By converting polyamines such as spermine, spermidine, and putrescine, the tumor growth was stifled. Moreover, the choice of the weaker ester bond between HA and bromo-butyric acid modified cholesterol (CH_Br) allowed a rapid release of BSAO [17]. This approach showed that the integrity of a given enzyme immobilization platform, as characteristic of the designable bonding types at play in a biopolymer system, can control the rate of enzyme and/or drug expression and release respectively. Freeze-thawing and freeze-drying storage processes further allowed for the NH-BSAO samples to maintain their specific activities to 100 and 80% of their initial values. Supportive in vitro analysis of human melanoma cancer cells revealed that BSAO post-immobilization successfully caused a cytotoxic effect on the cells equivalent to that of free BSAO, with such an effect also being accompanied by a negative correlation between cell viability and polyamine concentration [17].

### 3.2. Tissue Engineering and Wound Healing

**Figure 5:**
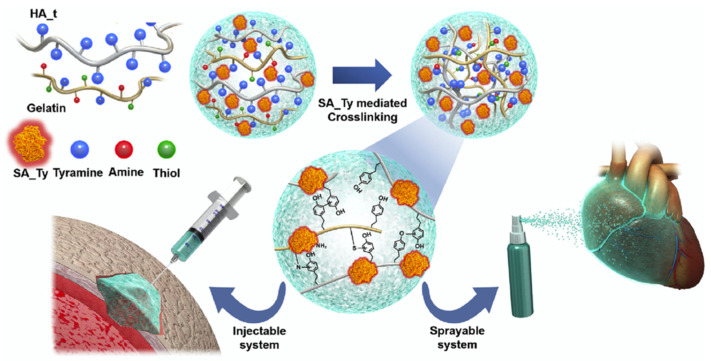
Visualization of the development of an HA-based, injectable and sprayable formula to be applied in tissue engineering as a post-surgical tissue adhesive and encouragement for cellular recruitment to damaged tissue. Reprinted with permission from ref. [18]. 2018 Elsevier.

The porous nature and structure of a HA-based hydrogel formed through crosslinking has proven imperative when scaffolds formation for cells and tissue growth as well as when regenerative processes were considered [69]. Further, the ability of the HA scaffold to swell was also shown to be critical for maintaining tissue viability [69], while its crosslinking capability conferred changes in gel stiffness [70], all to aid in HA’s implementation in tissue engineering and wound healing. Complementary, enzymatic integration was shown to aid in the healing itself or provide a bio-fructuous environment that promoted functionality and viability. For instance, Kim et al., produced a HA hydrogel with adhesive properties to be used as a surgical glue capable of promoting cell delivery and recruiting (Figure 5). HA’s biocompatibility, biodegradability, hydrophilicity, and ability to crosslink with proteins, formed a plausible network for solidly binding to wet environments before being replaced with repaired tissues. The first step of the gel forming procedure relied on the extraction and amplification of the tyrosinase gene from *Bacillus megaterium* (BM_Ty), *Agaricus bisporus* (AB_Ty), and *Streptomyces avermitilis* (SA_Ty) respectively, followed by the addition of crosslinker tyramine to the carboxyl group of HA, with the use of 1-ethyl-3-(3-dimethylaminopropyl) carbodiimide (EDC)/N-hydroxysuccinimide (NHS) coupling to result in a HA-tyramine conjugate (HA_t). Resulting tyrosinase was added to crosslink HA_t and gelatin. It was found that the addition of gelatin encouraged phenol-phenol, amine-tyrosine, and thiol-tyrosine coupling, while addition of NaCl decreased the viscosity of the hydrogel to that of a sprayable liquid [18]. Moreover, it was found that HA facilitated direct “molding” to the tissue and increased reactivity benefits as resulted from HA-enzyme complexes integration, respectively [18].

Following activity analysis of and comparisons among BM_Ty, AB_Ty, and SA_Ty, SA_Ty was found to be the most usable form of tyrosinase for crosslinking HA_t and gelatin, with resulting complex allowing for high catalytic activity, speed of reaction, and increased substrate specificity respectively. The improvement was due to increased substrate accessibility to the active site [18] to result in an optimal hydrogel composition of 3% (*w*/*v*) gelatin powder, 1% (*w*/*v*) HA_t, and 200 nM of SA_Ty enzyme respectively. Further, it was demonstrated that if the enzyme concentration was increased beyond the above, a high density of crosslinking-induced gelation occurred, and the solution grew too viscous to allow for spraying ability and injectability, the two specific administration routes tested in the authors’ approach. Lastly, when the conjugates were applied to mouse skin tissue ex vivo with degradation and biocompatibility of the hydrogel in vivo, negligible cytotoxicity as well as minimum effects on immune-related inflammation were observed, with the gel maintaining its mechanical properties as desired by a commercial surgical glue with activity being maintained through one week’s time [18].

A biocompatible scaffold hydrogel was developed using HA to successfully immobilize active trypsin and collagenase enzymes in a matrix of stem cells to help assist with migration of healing-promoting cells. Glial progenitors (GPs) and mesenchymal stem cells (MSCs) served as examples, with transgenic mice being used as model systems [19]. Analysis showed that the activity and viability of the HA-enzyme hydrogels were 60 and 28% of that of free enzyme activity, respectively, and that stem cells embedded in the HA-enzyme matrices also retained their survivability. The use of enzymes allowed for partial digestion of the connective tissue thus demonstrating increased efficiency in healing. Disadvantages to the injectable hydrogel however included a low enzymatic activity within a week of assembly, with collagenase activity decreasing to 33.3% of its initial one at Day 7 while immobilized trypsin’s activity decreased to 6.7% of its initial rate. Though HA proved advantageous in multiple ways including the enablement of MSC viability within this scaffold design, increasing HA concentration diminished the immobilized GP cell survival capability presumably due to increased hydrogel stiffness and thus induced limitation in substrate availability at the enzymatic active site [19].

Lastly, HA-enzymes-based systems were used in cartilage repair; for this, a collagen-HA hydrogel (Col-HA) served as a scaffold for loading bone marrow mesenchymal stem cells (BMSCs). Enzyme-catalyzed crosslinking limited the instability as well as the cytotoxicity of reaction products and agents, respectively. By modifying collagen and HA with tyramine and by applying a hydrogen peroxide/horseradish peroxidase (HRP) crosslinking through oxidative coupling of the collagen and HA, a fully biocompatible and sufficiently stable cartilage scaffold was created. The scaffold also allowed stem cell attachment and proliferation, and displayed adequate solubility for gelation, and physicochemical strength. Moreover, when different mass ratios of Col to HA were used (i.e., 100:0, 80:20, 60:40, and 50:50 termed ${\mathrm{Col}}_{5},{\;\mathrm{Col}}_{4},{\;\mathrm{Col}}_{3},{\;\mathrm{and}\;\mathrm{Col}}_{2.5}$ respectively) the rate of degradation was correlated with articular cartilage recovery. Applicable BMSC density was also observed following a week of in vitro fluorescence testing. It was found that the hydrogel enabled increased cell survival and proliferation rates. Further, in vivo analytical comparisons between concave defects untreated, treated with the Col-HA hydrogel, and treated with the BMSC-loaded hydrogel respectively, showed that while the control group could not self-heal, the hydrogel itself could fill in the defect, though with a fibrocartilage rather than desired cartilage such as what was found in the surrounding area. Lastly, the BMSC-Col-HA was shown to effectively fill in the defect with hyaline-cartilage-like tissue and allowed for proper incorporation within the existing cartilage environment [20].

### 3.3. Biosensing

Implantable biosensors rely on transduced electrical or optical signals induced by analyte-specific, biological or chemical reactions that occur at the interface of a bioreceptor component, such as an enzyme [71,72]. A considerable component to a given biosensor system is the electrode where a readable signal can be formulated for ultimate conversion and processing into a visualizable display to be initiated in conjunction with the initial analyte presence/concentration [21,71]. A common issue for implantable bioelectrodes, however, is the body’s natural response to foreign implantation which is known to consist of protein and cell adhesion onto the electrode [73] as well as scar tissue formation at the sensor side [74], all shown to inhibit biosensor performance and decrease its sensing functionality [21,73,74]. An electrochemical bioelectrode was assembled using HA and polydopamine (PDA) deposited onto an indium tin oxide electrode, with HA to attribute anti-fouling qualities to the electrode interface while also permitting for maintenance of electrical properties of the sensing complex respectively. The anti-fouling quality was likely due to the hydrophilic nature of HA which caused it to absorb and maintain considerable moisture around the electrode, while PDA allowed for increased immobilization through entrapping of HA molecules. Among the benefits resulting from such sensor electrode modification, authors notes a decrease of undesired protein adsorption, fibroblast adhesion, and scar tissue formation [21].

Complementary, Cabral et al., showed that HA hybridized with carbon nanotubes (CNTs) could be used for sensing of hepatitis B core protein antibody (Anti-HBc) marker to aid in the determination of hepatitis B virus (HBV) infection. The HA-CNT film consisted of HA chains wrapped around the circumference of the nanotubes; Anti-HBc served as a proof-of-concept representation of how a similar HA and biocatalyst-based sensor could be used. As a globally leading health issue and cause of death, spreading of HBV via blood transfusion is a major concern of health professionals [23,75]. The marker tested was envisioned to increase screening at blood banks, as anti-HBc can be present for the entirety of an individual’s life. CNTs met the demands of HA gelation required for the matrix’s stability as a viable platform for HBc immobilization as shown by scanning electron microscopy (SEM) [23], which further led to stable HA-induced dispersion of CNTs and easy HA-CNT complex-induced arrested phase separation [76]. The resulting gels were biocompatible, electrically conductive, and applicable for protein delivery via electrical stimulation. The CNT modification allowed for a more stable immobilization of HBc due to an enhancement of electron transfer; following the addition of the HA complex onto the electrode, there was about a 75.7% increase in the electroactive surface area. Furthermore, the electron transfer-based sensors were stable upon electrode immobilization [23].

**Figure 6:**
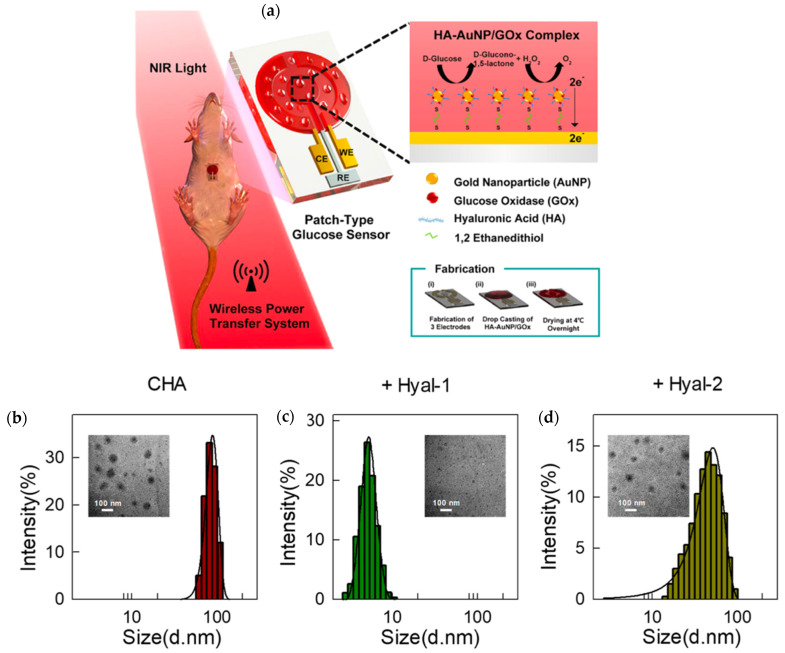
(**a**) Fabrication and application of a wireless glucose sensor used to test sweat for glucose concentration and consisting of an HA-AuNP/GOx complex casted on an electrode surface. Reprinted with permission from ref. [22]. 2019 American Chemical Society. (**b**–**d**) TEM imaging and size distribution corresponding to a 10 μg/mL solution of CHA without the addition of any hyaluronidase, CHA with 20 μg/mL Hyal-1, and CHA with 20 μg/mLHyal-2, respectively. Reprinted with permission from ref. [24]. 2019 American Chemical Society.

Considering the above, as well as the ability to integrate enzymatic transformation of specific substrates, products, and signals respectively, applications of enzymes and HA as biosensors have gained considerable interest for the detection of various analytes within a solution or a body sample. Placing enzymes within synthetic microenvironments, as characteristic of immobilization processes, was for instance shown to oftentimes increase their stability and reusability, qualities greatly desired for a biosensor [11,77,78,79,80]. Kim et al., developed a glucose sensor using hyaluronate (the salt form of HA), AuNPs, GOx, and an integrated circuit chip; the sensor was intended to process sweat glucose data collected at its interface [22]. HA’s integration inhibited the interference of otherwise detrimental molecules including lactic acid and L-ascorbic acid. As a result of these benefits, accurate and specific measurement, non-invasiveness, stability, sensitivity, and feasibility were enabled when monitoring continuous diabetes detection in mice. As shown in Figure 6a, the complex consisted of enzyme and HA-coated AuNPs attached to the surface of a functional electrode via binding of each element with 1,2, ethanedithiol [22].

The designed HA-AuNP/GOx complex, maintained 78% activity for about 4 days under repeated use in comparison to a complete loss of GOx coating and corresponding activity following only 2 days of operation. Further, stability testing showed that the addition of HA-AuNP to the enzyme system increased the percentage of initial current that was maintained from 29.6 to 98.1%, before and after HA-AuNP integration, respectively. In comparison to the sensors coated with GOx and AuNP/GOx, the HA-AuNP/GOx sensors possessed the lowest detection limit at 0.5 mg ${\mathrm{dL}}^{-1}$. Moreover, the HA-AuNP/GOx electrode coating saw improvement in reusability when compared to sensors coated with GOx alone. Specifically, it was shown that the conjugation of HA and AuNPs allowed a stable platform for GOx immobilization that inhibited enzyme degradation and improved glucose sensing when compared to GOx and AuNP/GOx sensor coatings alone. With the known blood glucose levels of 179, 288, and 374 mg/dL, the output codes of the HA-AuNP/GOx glucose sensor were 289.7, 494.0, and 602.9 respectively, suggesting a direct and accurate positive correlation between glucose level and biosensor reading. Because of the fast response time of only 5 s, the sensor was nearly real-time, another major benefit for diagnostics or glucose monitoring [22].

Due to their ability to break down HA, Hyal-1 and Hyal-2 (hyaluronidase enzymes) were investigated by Li et al., as physiological analytes to a HA-based fluorescent sensing platform. Specificity was both desired and achieved for the detection of Hyal-1 over Hyal-2 because of its prevalence in tumor sites, more precisely found in bladder, prostate, and breast cancer patients. The biosensor design integrated HA functionalized with cholesterylamine (CHA) to form and entrap RNA-binding fluorophores (RBFs); when Hyal-1 was present, the CHA nano-assembly was degraded into small fragments to unveil the fluorophores and thus offer a fluorescence-attributed detection of Hyal-1 containing cancerous tissues. The specific Hyal-1-induced disassembly of CHA and the implied application for fluorescence imaging of cancer sites is shown in Figure 6b, as most CHA particles were below 10 nm in diameter as compared to distributions entirely greater than this 10 nm mark seen in CHA alone and CHA with Hyal-2. Additionally, RBF was able to bind to cytoplasmic RNA to further amplify the recorded fluorescence signal thus aiding in the ease of detection. In comparison to other intracellular sensing systems, the use of fluorescence detection was less invasive, displayed high resolution, and had the ability to show results in real-time [24].

Authors also showed that in the development of an innovative hyaluronidase identification system, self-assembled, amphiphilic CHA shells encompassing and stifling the fluorescence of RBFs were more likely broken apart by Hyal-1 than by Hyal-2. Small levels of Hyal-1 were discernable by the RBF@CHA systems, as characterized by a detection limit of 1 × $10^{-5}$ μg ${\mathrm{mL}}^{-1}$ and a linear detectable concentration range between 0 and 0.01 μg ${\mathrm{mL}}^{-1}$. Meanwhile, presence of Hyal-2 did not present a clear fluorescence amplification of the RBF@CHA assemblies, as expectedly characteristic of an enzyme-degraded composite. In addition to Hyal-2, Hyal-1 specificity analyses revealed that RBF@CHA was found not to be affected by biomolecules such as $H_{2}O_{2}$, biothiol glutathione, cathepsin, trypsin, thrombin, lysozyme, ribonuclease, and galactosidase respectively. Moreover, the RBF@CHA showed feasible biocompatibility for both cellular uptake and lack of cytotoxic effects when studied in living HeLa cells [24].

## 4. Challenges and Perspectives for HA-Enzyme Applications: A Complementary Market Analysis

Above examples demonstrate targeted strategies based on HA-enzyme conjugates for applications as drug nanocarriers, scaffolds to control compatibility and adherence within the body, to have sufficient stability, entrapment capabilities, and to allow for user-controllability with ease of implementation, or as stable, real-time, and biocompatible biosensors. From facilitating the accumulation at the tumor site, to helping receive increased penetration through cancerous tissue, and ultimately by allowing effective drug delivery design strategies, HA-enzyme conjugates have not only demonstrated increased ease of implementation but further, increased preservation of biocompatibility as well as superior degree of biodegradability [20], all while being reproducible at manufacturing and cost efficient.

It is envisioned that the benefits of the enzymatic technologies integration with HA are only to increase. Specifically, global enzyme market potential anticipates a 7.1% CAGR between 2020 and 2027 [81]. Moreover, if in 2019, the global enzyme market was worth USD 9.9 billion it is now estimated that such market will reach USD 17.17 billion by the end of 2027 [81]. Furthermore, in addition to the overarching industry of biocatalysis where most of the market increases were initially noted, recently there has been an increase in the demand for immobilized enzyme technologies to be applied in areas such as the pharmaceutical, environmental, food, diagnostics, and biotranformation industries, al such desirable applications resulting from the advantages that such systems implementation provide mainly their prolonged shelf-life and improved recyclability capacity especially relative to free enzymes [82]. Such increase in enzymes’ and immobilized enzymes’ markets are complemented by the rapid growth of their applicable industries namely the market of biomaterials for drug-delivery grew from roughly USD 178.8 billion in 2015 to USD 227.3 billion in 2020 [83].

Moreover, the drug delivery and biomaterial markets are expected to continue a parallel growth, along with that of other biomedical fields such as tissue engineering, the global medical enzyme market, and the hyaluronic acid market. For instance, in 2019, the tissue engineering market reached USD 25.4 billion, and over the span of 2017–2022, the market is on the track to increase 17.22% in CAGR [84]. Complementarily, the biomaterials market is particularly successful with a growing demand for products such as medical implants to be utilized as the primary scaffold of tissue-engineered heart valves [85], as well as nano-, micro-, and macroscale drug systems developed for cancer immunotherapy and antitumor T-cell immunity [86]. To add in, the biomaterials sector is poised to increase from a worth of USD 105 billion in 2019 to USD 207 billion in 2024, which reaches a 14.4% CAGR [87]. Meanwhile, the global medical enzyme market is estimated to grow at a CAGR of 6.7% in the years spanning from 2021–2028, also projected to increase from USD 4.0 billion in 2020 to USD 6.7 million in 2028 [88]. As a key component to several of the recent biomedical implementations of enzyme immobilization discussed, the HA market has also seen a sizeable growth rate of 15% per annum since the market was established in 1997. It is noted that HA has not established a particularly strong market in Europe but is seemingly successful in the US [89], likely explainable by the country’s comparatively high interest in cosmetic enhancement. As such, the HA market is estimated to reach USD 15.4 billion by the year 2025, which is a considerable increase from its previous value of USD 7.2 billion in 2016 [90].

These listed markets analysis have seen many contributing social and economic advantages in terms of renewable sourcing, high reaction efficiency, low production costs, long shelf-life, and reusability respectively [82]. For example, biotechnologies are centered around materials such as enzymes and HA, both ethically sourced from growable biological matter and able to be viably transposed to other biological entities in a non-species-specific manner. When compared to free enzymes, immobilized enzymes have properties that make them more catalytically active, substrate-specific, and easily separated and recovered following catalysis [91]. This allows a wide breadth of efficiency and increased sustainability for the immobilized catalysts and even consideration for new technologies implementation, thus making them more desirable as points of investment and lucrativeness in the business world as well as in science. When comparing the cost effectiveness of free and immobilized enzymes for instance, a mathematical model was created and concluded that the immobilized enzymes see a 60% reduction in costs of production for instance [92]. This decrease in overhead costs is predicted to allow markets to sustainably produce biomedical materials and devices that utilize immobilized enzymes, as the high sourcing costs of enzymes can be offset by the ability to reuse those catalytic feedstocks more than once.

However, HA-enzyme conjugates integration, while promising in terms of both bio-related benefits and market potential, continues to still be limited by elements such as the type of administration route for implantation [15], loss of enzyme activity following storage processes such as freeze-thawing and freeze-drying [17], and/or the decrease in enzyme stability especially at certain pH values [93]. Additionally, lack of knowledge of the span of effects HA can have in vivo [19], especially considering that an HA-based platforms are often fragile, with changes in its structural integrity not only to decrease enzymatic activity and impact the stability characteristics of a HA-based gel used as scaffold [18] but also to possibly lead to the production of by-products and free radicals that could induce inflammation and possible signs of cytotoxicity [70] especially when considering the shelf-life of such conjugates, since prolonged enzymatic activity is required to make described gels and scaffolds be viable for storage, transportation, and use in the medical industry [19] respectively.

The main objectives of the markets that were discussed are profitability, efficiency, and most importantly, sustainability. We envision that the next generation of enzyme-HA conjugates need to be controlled, integrated, and optimized for synthetic applications. In doing so, the potential market growth in each of the mentioned sectors as well as commercial development can be fulfilled. Continued development of enzyme and HA-based technologies is vital for the evolution of our health systems as well as energy and consumption needs on a global scale. Therefore, with numeric proof of economic aptitude in addition to potential for positive societal impact, funding for industry-scale projects is highly feasible in the near-future.

## 5. Conclusions

The application of enzymes for catalytic processes has become an impactful sector in biofuels, plastic and chemical waste management, detergents, textiles, and food production and preservation. Within these industries, biocatalysis provides the enablement of specific and desirable reactions at the core technological advancements, as related to the rate and yield of product formation, all in a green, self-sustainable and specific manner.

There are limitations, however, as their systematic benefits are accompanied by their sensitivity to synthetic environments, with such caveats leading to their denaturation and their loss of catalytic activity in temperatures and acidic conditions. Because so many developments in biotechnology, particularly within the biomedical field, require extreme conditions upon execution and/or storage, there is frequently a necessity for a method in which an enzyme’s stability can be enhanced relative to that of its free form. Explored here was the technique of enzyme immobilization, wherein enzymes are chemically or physically linked or encapsulated within a structure that encourages enzyme stability and increases its shelf-life while maintaining its functionality and product yield.

Focus was placed on enzyme integration with HA. HA-enzyme conjugates applications in drug delivery, as scaffolds and as biosensors were thus discussed. With large consideration to biomedicine, a given immobilization platform must encompass the values of a stable scaffold that can be successfully conjugated with enzymes, yet it must also have intrinsic qualities pertaining to biocompatibility and in some cases, instrumental mechanisms such as biodegradation and targeted delivery. HA advantages as a highly modifiable polysaccharide were further analyzed from the perspective of the global market implementation in biomedicine. Thus, evaluating enzyme market potential and implementation capacity are a matter of users and their ability to control or envision how products and byproducts are to be integrated or separated in an efficient and controlled fashion.
